# Comparison of Magnifying Endoscopy with Blue Light Imaging and Narrow Band Imaging for Determining the Invasion Depth of Superficial Esophageal Squamous Cell Carcinoma by the Japanese Esophageal Society’s Intrapapillary Capillary Loop Classification

**DOI:** 10.3390/diagnostics11111941

**Published:** 2021-10-20

**Authors:** Waku Hatta, Tomoyuki Koike, Yohei Ogata, Yutaka Kondo, Nobuyuki Ara, Kaname Uno, Naoki Asano, Akira Imatani, Atsushi Masamune

**Affiliations:** 1 Division of Gastroenterology, Tohoku University Graduate School of Medicine, 1-1 Seiryo-machi, Aoba-ku, Sendai 980-8574, Japan; tkoike@rd5.so-net.ne.jp (T.K.); gmaps177@gmail.com (Y.O.); kaname@wa2.so-net.ne.jp (K.U.); asanon@med.tohoku.ac.jp (N.A.); aimatani@med.tohoku.ac.jp (A.I.); amasamune@med.tohoku.ac.jp (A.M.); 2 Division of Gastroenterology, Tohoku Rosai Hospital, 4-3-21 Dainohara, Aoba-ku, Sendai 981-8563, Japan; kyutaka@tohokuh.johas.go.jp; 3 National Hospital Organization Sendai Medical Center, Department of Gastroenterology, 2-11-12 Miyagino, Miyagino-ku, Sendai 983-8520, Japan; nobuara@med.tohoku.ac.jp

**Keywords:** esophageal squamous cell carcinoma, invasion depth, blue light imaging, narrow-band imaging

## Abstract

Blue light imaging (BLI) and narrow-band imaging (NBI) are two modalities that enable narrow-band light observation. We aimed to compare the diagnostic ability of magnifying endoscopy with BLI (ME-BLI) and NBI (ME-NBI) for determining the invasion depth of superficial esophageal squamous cell carcinoma (SESCC) by the Japanese Esophageal Society’s intrapapillary capillary loop (IPCL) classification. We enrolled 81 patients between 2014 and 2018, and the still endoscopic images for diagnosing the invasion depth at the same part in ME-BLI and ME-NBI were registered. Two blinded investigators reviewed them and diagnosed the invasion depth by the IPCL classification. Subsequently, the diagnostic yields in two modalities were compared. The overall accuracies for the invasion depth by the IPCL classification in ME-BLI and ME-NBI did not differ significantly (67.9–71.6% vs. 72.8–74.1%). In the analysis based on the invasion depth, the sensitivities and positive predictive values in tumors invading the muscularis mucosa or submucosa ≤200 µm were low (23.1–30.8% and 16.7–25.0%, respectively) in both modalities. In conclusion, the diagnostic ability for determining the invasion depth of SESCC by the IPCL classification was relatively similar in ME-BLI and ME-NBI, but diagnosis by magnifying endoscopy alone might not be satisfactory.

## 1. Introduction

Esophageal cancer is the seventh leading cause of the cancer incidence and the sixth leading cause of cancer death [1], and esophageal squamous cell carcinoma accounts for 84% of cases with esophageal cancers [2,3]. Endoscopic resection (ER) is a minimally invasive treatment method for superficial esophageal squamous cell carcinoma (SESCC) without lymph node metastasis [4], and favorable long-term outcomes have been reported [5,6,7]. According to the latest Japanese guidelines [8], ER is recommended for tumors confined to the epithelium or lamina propria mucosa (cT1a-EP/LPM) or those invading into the muscularis mucosa or submucosa ≤200 μm (cT1a-MM/T1b-SM1) when the tumors are not circumferential. On the other hand, surgical resection or chemoradiotherapy is recommended for those invading into the submucosa >200 µm (cT1b-SM2). Thus, predicting the invasion depth is crucial for the appropriate treatment decision.

For preoperative diagnosis of the tumor invasion depth, magnifying endoscopy as well as endoscopic ultrasonography and non-magnifying endoscopy are recommended [8]. The intrapapillary capillary loop (IPCL) patterns are the major determinants for tumor invasion depth in magnifying endoscopy [9], and the classifications developed by Inoue et al. [9,10] and Arima et al. [11] had been used. Recently, the Japan Esophageal Society (JES) developed a simplified magnifying endoscopic classification for estimating invasion depth [12], and this is now widely applied. In this classification, IPCLs are classified into type A, corresponding to noncancerous lesions and lacking severe irregularity, and type B, corresponding to cancerous lesions and exhibiting severe irregularity. Type B vessels were subclassified into B1, B2, and B3 for the diagnostic invasion depth of SESCC. Narrow-band imaging (NBI) has been reported as a useful method for the detection and diagnosis of SESCC [13]. Furthermore, magnifying endoscopy with NBI (ME-NBI) in the JES’s IPCL classification is noted for having relatively good accuracy in predicting the invasion depth of SESCC [14].

Blue light imaging (BLI) is a novel image-enhanced endoscopy with two light sources that enable narrow light observation [15]. The recognition of SESCC using BLI-bright, which produces a brighter and narrow light image, is reported to be more efficacious than NBI [16]; however, the usefulness of magnifying endoscopy with BLI (ME-BLI) for evaluating the invasion depth of SESCC by the JES’s IPCL classification has not been fully clarified. Hence, this study aimed to compare ME-BLI with ME-NBI. This study also evaluated whether the diagnosis for determining the invasion depth of SESCC by magnifying endoscopy alone is reliable. 

## 2. Materials and Methods

### 2.1. Study Design

This is a comparative study using ME-BLI and ME-NBI, and the patients were prospectively enrolled. The study was performed in accordance with the Declaration of Helsinki and the ethical guidelines for medical and health research involving human subjects in Japan. The study protocol was approved by the Ethics Committee of the Tohoku University Hospital (2014-2-028-1; approved on 27 May 2014) before the recruitment of patients. All participants provided written informed consent prior to enrollment in the study.

### 2.2. Study Population

The eligibility criteria were patients who had a SESCC diagnosed by biopsy and those 20 years or older and with 0–2 in Eastern Cooperative Oncology Group performance status. The exclusion criteria were (1) patients with a history of surgery, chemotherapy or radiation therapy against head and neck or esophageal cancers; (2) those who had esophageal stenosis or esophageal varices; (3) those with a history of chemotherapy or radiation therapy against other cancers; (4) those who were pregnant, within 28 days after childbirth, or breastfeeding; (5) those who had mental diseases and were judged as having difficulty in participating in this study.

### 2.3. Study Procedure

In the examination stage, the ME-BLI examination, followed by the ME-NBI examination, for SESCC was performed by either of two expert endoscopists (WH, TK). The interval between the two examinations was within one week. The ME-BLI examination was conducted using an EG-L590ZW endoscope and the LIGHTEO endoscopic systems consisting of a VP-4450HD and an LL-4450 light source (Fujifilm Co., Ltd., Tokyo, Japan), and still ME-BLI images of whole lesions, as far as possible, were acquired. The image and color enhancement modes were B6 and C1, respectively. Just after the examination, a still ME-BLI image was selected in each case via discussion by two endoscopists (WH, TK) and the image was registered. In this process, when IPCL without a loop formation and/or large caliber vessel was suspected in the ME-BLI mages, these images were selected for review. Subsequently, the ME-NBI examination was conducted using a GIF-H260Z endoscope and EVIS LUCERA SPECTRUM system or EVIS LUCERA ELITE system (Olympus Medical Co., Ltd., Tokyo, Japan), and the image and color enhancement modes were A8 and 1, respectively. A still endoscopic image for the same part as that examined by ME-BLI was registered in each case. In each examination, magnifying endoscopic observation was performed with the maximum magnification (ME-BLI, 135-fold; ME-NBI, 80-fold) as far as possible.

**Figure 1:**
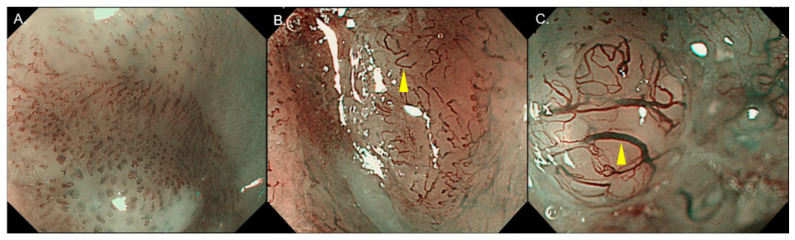
Typical images of B1, B2, and B3 vessels in the JES’s IPCL classification in ME-NBI. (**A**) B1 vessel. (**B**) B2 vessel (yellow arrow). (**C**) B3 vessel (yellow arrow). JES, Japan Esophageal Society; IPCL, intrapapillary capillary loop; ME-NBI, magnifying endoscopy with narrow band imaging.

**Figure 2:**
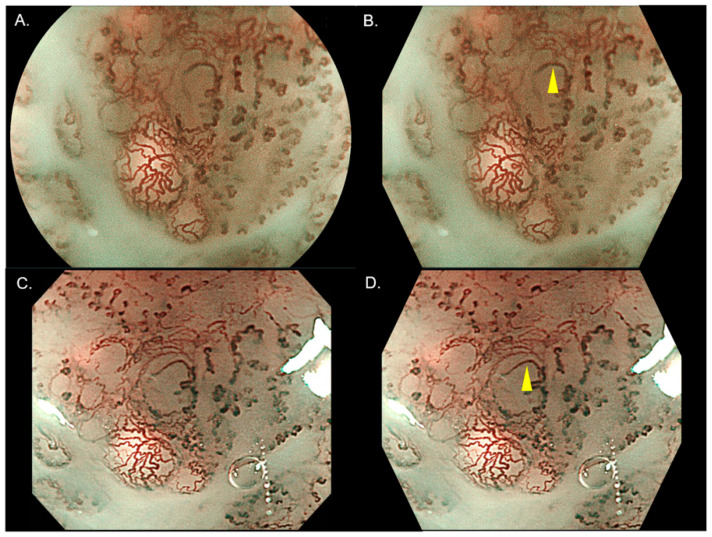
Standardization of the ME-BLI and ME-NBI images for review. ME-BLI (**A**) and ME-NBI (**C**) images were standardized to the same shape (**B**,**D**) to reduce the bias. The calibers of some B2 vessels in ME-BLI (yellow arrows) appeared larger than those in ME-NBI. ME-BLI, magnifying endoscopy with blue light imaging; ME-NBI, magnifying endoscopy with narrow band imaging.

In the review stage, two blind investigators (YK, NA) diagnosed the invasion depth of SESCC by reviewing the still images of ME-BLI and ME-NBI. These investigators were expert endoscopists familiar with ME-BLI and ME-NBI and had treated over 100 cases with SESCCs by ER. The investigators confirmed several typical images of the JES’s IPCL classification before the start of the review (Figure 1A–C). To reduce bias, the endoscopic images were standardized to the same shape (Figure 2A–D) and numbered randomly before the start of the review. At one week after the review, two investigators reviewed the still images and diagnosed the invasion depth again.

### 2.4. The Definition of the Invasion Depth of SESCC Based on the Magnifying Endoscopy

According to the JES’s IPCL classification [12], the type of vessel for predicting the invasion depth of SESCC was classified into three categories as follows: B1 (Figure 1A), with severe irregularity or high dilatation of IPCL with a loop-like formation, for cT1a-EP/LPM; B2 (Figure 1C), with severe irregularity or high dilatation of IPCL without a loop-like formation, for cT1a-MM/T1b-SM1; B3 (Figure 1C), with highly dilated irregular vessels in which the calibers appear to be more than three times that of usual B2 vessels, for cT1b-SM2 or deeper.

### 2.5. Pathological Diagnosis

The SESCCs were resected by endoscopic submucosal dissection. All specimens were cut into 2-mm-wide longitudinal slices after fixation in 10% buffered formalin before examination under hematoxylin-eosin staining and, if necessary, immunostaining. Pathological diagnosis was performed by expert pathologists according to the Japanese classification [17,18].

### 2.6. Outcome Measures

First, we compared the overall diagnostic accuracy of the invasion depth of SESCC by the JES’s IPCL classification between ME-BLI and ME-NBI. Second, the sensitivities, specificities, positive predictive values (PPVs), and negative predictive values (NPVs) for the diagnosis of the invasion depth of SESCC were compared between the two modalities. We also evaluated interobserver and intraobserver agreements of the diagnostic invasion depth by the classification in each examination of ME-BLI and ME-NBI.

### 2.7. Statistical Analysis

Continuous variables were expressed as the mean and standard deviation. Categorical variables were expressed as the frequency and proportion, and the two groups were compared using chi-square test. Interobserver and intraobserver agreements were estimated using a Cohen’s kappa coefficient, and the values of <0.20, 0.21–0.40, 0.41–0.60, 0.61–0.80, and 0.81–1.00 were considered to indicate poor, fair, moderate, good, and excellent agreement, respectively. Data were analyzed using SPSS version 23.0 for Windows software (IBM Corp., Armonk, NY, USA). All *p* values were two-tailed, and a *p* value of <0.05 was regarded as statistically significant for each test.

## 3. Results

### 3.1. Baseline Characteristics

Between November 2014 and July 2018, a total of 81 patients, comprising 67 males and 14 females, were enrolled in this study. The mean tumor size was 26.8 mm. The number of cases with pT1a-EP/LPM, pT1a-MM/T1b-SM1, and pT1b-SM2 were 64, 13, and 4 cases, respectively. The detailed characteristics of the enrolled patients are shown in Table 1. Both investigators accurately identified as ME-BLI or ME-NBI in endoscopic images of the same shape in all cases.

### 3.2. Diagnostic Performance in ME-BLI and ME-NBI

In investigator 1, the overall accuracies for diagnosing the invasion depth by the IPCL classification in ME-BLI and ME-NBI were similar (71.6% vs. 72.8%). In investigator 2, the overall accuracy in ME-BLI was a little lower than that in ME-NBI (67.9% vs. 74.1%), but the difference was not significant (*p* = 0.387). The detailed relationship between the IPCL classification and pathological invasion depth in ME-BLI and ME-NBI is shown in Table 2.

The sensitivities, specificities, PPVs, and NPVs for the invasion depth by the IPCL classification in two modalities are presented in Table 3. In pT1a-EP/LPM, the sensitivities were high with 81.3–84.4% in ME-BLI and 85.9–87.5% in ME-NBI, and the PPVs were also high. Conversely, the sensitivities and PPVs in pT1a-MM/T1b-SM1 were low in both modalities (sensitivity, 23.1–30.8%; PPV, 16.7–25.0%). Regarding pT1b-SM2, no cases had B3 vessels, possibly because the number of cases with pT1b-SM2 was limited.

### 3.3. Interobserver and Intraobserver Agreement

The kappa values for the interobserver agreement in ME-BLI and ME-NBI were good (0.60) and good (0.77), respectively. Those for the intraobserver agreement in these two modalities were good (0.66) and good (0.75), respectively, in investigator 1 and excellent (0.89) and good (0.78), respectively, in investigator 2. The detailed concordance between two investigators and that between the first and second reviews are shown in Table 4.

### 3.4. Different Diagnosis of the Invasion Depth by the IPCL Classification between ME-BLI and ME-NBI

**Figure 3:**
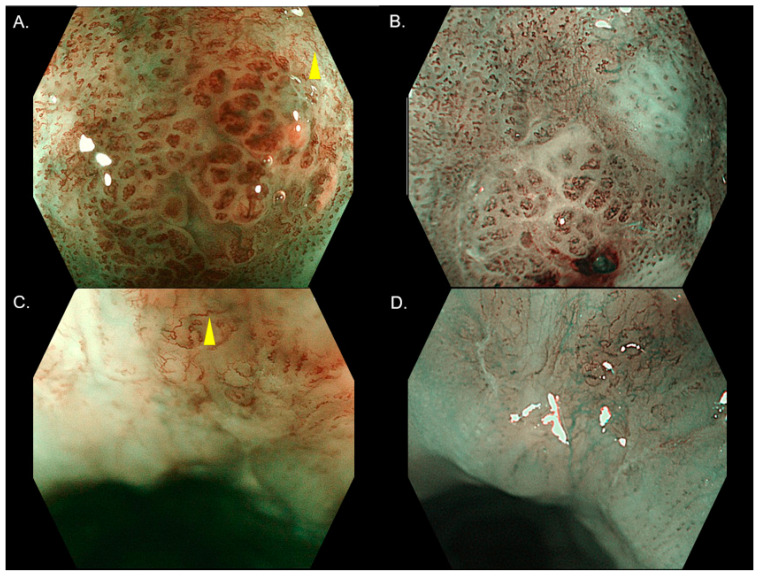
Cases with different diagnoses of the invasion depth between ME-BLI and ME-NBI. (**A**) ME-BLI image in case 1. (**B**) ME-NBI image in case 1. (**C**) ME-BLI image in case 2. (**D**) ME-NBI image in case 2. In both cases, two investigators diagnosed them as having B2 vessels (yellow arrows) in ME-BLI, whereas only B1 vessels were found by them in ME-NBI. ME-BLI, magnifying endoscopy with blue light imaging; ME-NBI, magnifying endoscopy with narrow band imaging.

There was a difference in the diagnosis of the invasion depth by the IPCL classification between ME-BLI and ME-NBI in 11 cases and 10 cases in investigators 1 and 2, respectively (Table 4). Although the calibers of IPCL in ME-BLI seemed to appear larger and thinner vessels were detected in ME-BLI (Figure 3A–D), the distributions of B1 and B2 vessels in ME-BLI and ME-NBI were similar for both investigators (Table 4). 

### 3.5. Misdiagnosed Cases in ME-BLI and ME-NBI

In ME-BLI, both investigators diagnosed as having B2 vessels in six cases with pT1a-EP/LPM, whereas they diagnosed as having only B1 vessels in eight cases with pT1a-MM/T1b-SM1. In ME-NBI, they diagnosed as having B2 vessels in five cases with pT1a-EP/LPM, while nine cases with pT1a-MM/T1b-SM1 cases were diagnosed as having only B1 vessels by both investigators. Both investigators diagnosed as having B2 vessels but no B3 vessels in three of four cases with pT1b-SM2. 

## 4. Discussion

NBI and BLI are two modalities that enable narrow-band light observation; however, the bandwidth and wavelength differ between the BLI and NBI systems. The bandwidth in BLI (2 nm) is much narrower than that in NBI (30 nm) [19,20]. Furthermore, BLI is made by a combination of 410 and 450 nm lights, whereas NBI is a technique by which spectral features are modified by narrowing the bandwidth of spectral transmittance using filters adjusted to both 415 and 540 nm [19,20]. These differences may affect the diagnostic ability for determining the invasion depth of SESCC and, thus, we conducted this comparative study.

This study revealed that ME-BLI and ME-NBI had relatively similar diagnostic yields. However, different diagnoses of the invasion depth between the two modalities appeared in several cases. As shown in Figure 3, IPCLs in ME-BLI appeared more brownish and their calibers were larger, and these differences might be partially due to differences in the bandwidth and wavelength between the two modalities. Furthermore, ME-NBI may have detected thinner vessels. However, according to the analysis of the relationship of the IPCL classification between the two modalities, the distributions of B1 and B2 vessels were similar in both investigators. Thus, the difference in the appearance between ME-BLI and ME-NBI might be tiny for diagnosing the invasion depth of SESCC.

The sensitivities and PPVs in the present study were lower than those in previous reports [12,21]. In particular, those of pT1a-MM/T1b-SM1 were much lower (sensitivity, 23.1–30.8%; PPV, 16.7–25.0%). One of the reasons might be that only one image of each modality was selected in each case. Furthermore, the narrow area of B2 vessels might have affected the results. In this study, when B2 vessels were found, the lesion was diagnosed as cT1a-MM/T1b-SM1. However, a recent study revealed that a B2 vessel area <6 mm in diameter was an independent risk factor for overdiagnosis of the invasion depth [22]. Another recent study revealed that, although the PPV in pT1a-MM/T1b-SM1 was 33.3%, it increased to 77.3–87.0% by using the B2-narrow/broad subclassification with a cut-off value of 4 mm in diameter [23]. Thus, a wide area (e.g., 4 or 6 mm in diameter) of B2 vessels might be an indicator for a deeper invasion depth in SESCC. Furthermore, based on our results, endoscopic diagnosis of the invasion depth by magnifying endoscopy alone might not be satisfactory. However, a previous study demonstrated that the combination of white light imaging and ME-BLI/ME-NBI had higher accuracy for diagnosing the invasion depth of SESCC than each white light imaging, ME-BLI, and ME-NBI [21]. Thus, the combination of two modalities might be desirable for the diagnosis.

Some SESCCs with B2 vessels were diagnosed as pT1b-SM2. Such patients may have a risk of delay in receiving definitive treatment, such as esophagectomy or chemoradiotherapy (CRT). Although SESCCs with pT1b-SM2 have a risk of metastatic recurrence (23.6% in five years) when additional treatment is not received [6], a recent multicenter prospective study revealed the efficacy of the combination of ER and selective CRT, which is comparable to the efficacy of esophagectomy [24]. Considering that ER is a minimally invasive treatment method and the selection of esophagectomy or CRT by overdiagnosis of the invasion depth may be a disadvantage, the selection of ER based on B2 vessels might be acceptable.

The present study has several limitations. First, although the patients were prospectively collected, endoscopic images were retrospectively analyzed in this study, which includes a potential bias. Second, this is a single-center study with a small number of cases. Third, this study selected a still image by two expert endoscopists in the ME-BLI examination in each case, followed by the acquisition of a ME-NBI image at the same part as that in ME-BLI. The endoscopists carefully observed the whole lesion in ME-BLI and, if B2 or B3 vessels were observed, this image was selected; however, this study design has a potential for selection bias. Furthermore, the selection of the part for review was based on ME-BLI but not on ME-NBI. Fourth, the number of cases with pT1b-SM2 was so small and only one case showed B3 vessels, which led to an imbalance in the three categories of tumor invasion depth. Lastly, the invasion depth of SESCC was diagnosed by only two investigators. Furthermore, although these investigators were expert endoscopists and they confirmed several typical images of the JES’s IPCL classification before starting the review, it is difficult to certify whether the investigators adequately applied the classification, and their interpretation of the classification might have affected the results. However, artificial intelligence (AI) may overcome this issue. Recently, the application of AI systems showed a favorable performance for detecting and diagnosing the invasion depth of SESCC [25,26]. Moreover, robotic endoscopy with advanced functionalities has been developed and mastered [27,28]. The implementation of AI systems in novel robotic devices, combined with remote telehealth services, will contribute to the more reliable and universal application of the IPCL classification in diagnosing the invasion depth of SESCC.

In conclusion, we demonstrated relatively similar diagnostic ability of the invasion depth of SESCC by the JES’s IPCL classification between ME-BLI and ME-NBI. Despite these modalities having different bandwidths and wavelengths, this difference might be tiny for the diagnosis. Furthermore, the diagnostic yield, especially based on B2 vessels, was not sufficient. Thus, the endoscopic diagnosis of the invasion depth of SESCC by magnifying endoscopy alone might not be adequate, regardless of ME-BLI or ME-NBI.

## Figures and Tables

**Table 1:** The baseline characteristics of the enrolled patients.

	SESCC Patients (*n* = 81)
Age (y), mean (SD)	68.4 (9.4)
Sex, *n* (%)	
Male	67 (82.7)
Female	14 (17.3)
Tumor location, *n* (%)	
Upper part	8 (9.9)
Middle part	51 (63.0)
Lower part	22 (27.2)
Tumor invasion depth, *n* (%)	
pT1a-EP/LPM	64 (79.0)
pT1a-MM/T1b-SM1	13 (16.0)
pT1b-SM2	4 (4.9)
Tumor size (mm), mean (SD)	26.8 (17.4)

SESCC, superficial esophageal squamous cell carcinoma; SD, standard deviation; T1a-EP/LPM, tumors confined to the epithelium or lamina propria mucosa; T1a-MM/T1b-SM1, tumors invading into muscularis mucosa or submucosa ≤200 μm; T1b-SM2, tumors invading into submucosa >200 µm.

**Table 2:** The relationship between the JES’s IPCL classification and pathological invasion depth in ME-BLI and ME-NBI.

Pathological Invasion Depth	JES’s IPCL Classification					
	ME-BLI			ME-NBI		
	B1	B2	B3	B1	B2	B3
Investigator 1						
pT1a-EP/LPM	54	10	0	55	8	1
pT1a-MM/T1b-SM1	9	4	0	9	4	0
pT1b-SM2	1	3	0	0	4	0
	Accuracy: 71.6%			Accuracy: 72.8%		
Investigator 2						
pT1a-EP/LPM	52	12	0	56	8	0
pT1a-MM/T1b-SM1	10	3	0	9	4	0
pT1b-SM2	1	3	0	0	4	0
	Accuracy: 67.9%			Accuracy: 74.1%		

JES, Japan Esophageal Society; IPCL, intrapapillary capillary loop; ME-BLI, magnifying endoscopy with blue light imaging; ME-NBI, magnifying endoscopy with narrow band imaging; pT1a-EP/LPM, tumors confined to the epithelium or lamina propria mucosa; pT1a-MM/T1b-SM1, tumors invading into muscularis mucosa or submucosa ≤200 μm; pT1b-SM2, tumors invading into submucosa >200 µm.

**Table 3:** Diagnostic ability for the invasion depth of SESCC by the JES’s IPCL classification between ME-BLI and ME-NBI.

	ME-BLI				ME-NBI			
	SE, %	SP, %	PPV, %	NPV, %	SE, %	SP, %	PPV, %	NPV, %
Investigator 1								
pT1a-EP/LPM	84.4	41.2	84.4	41.2	85.9	47.1	85.9	47.1
pT1a-MM/T1b-SM1	30.8	80.9	23.5	85.9	30.8	82.4	25.0	86.2
pT1b-SM2	0.0	100.0	–	95.1	0.0	98.7	0.0	95.0
Investigator 2								
pT1a-EP/LPM	81.3	35.3	82.5	33.3	87.5	47.1	86.2	50.0
pT1a-MM/T1b-SM1	23.1	77.9	16.7	84.1	30.8	82.4	25.0	86.2
pT1b-SM2	0.0	100.0	–	95.1	0.0	100.0	–	95.1

SESCC, superficial esophageal squamous cell carcinoma; JES, Japan Esophageal Society; IPCL, intrapapillary capillary loop; ME-BLI, magnifying endoscopy with blue light imaging; ME-NBI, magnifying endoscopy with narrow band imaging; SE, sensitivity; SP, specificity; PPV, positive predictive value; NPV, negative predictive value; pT1a-EP/LPM, tumors confined to the epithelium or lamina propria mucosa; pT1a-MM/T1b-SM1, tumors invading into muscularis mucosa or submucosa ≤200 μm; pT1b-SM2, tumors invading into submucosa >200 µm.

**Table 4:** Interobserver and intraobserver agreements for diagnosing the invasion depth of SESCC by the JES’s IPCL classification.

Interobserver Agreement							
Investigator 1				Investigator 2			
	ME-NBI				ME-NBI		
ME-BLI	B1	B2	B3	ME-BLI	B1	B2	B3
B1	59	5	0	B1	59	4	0
B2	5	11	1	B2	6	12	0
B3	0	0	0	B3	0	0	0
Kappa value	0.59			Kappa value	0.63		
**Intraobserver agreement (investigator 1)**							
**ME-BLI**				**ME-NBI**			
	**Second Review**				**Second Review**		
First review	B1	B2	B3	First review	B1	B2	B3
B1	59	5	0	B1	62	2	0
B2	2	12	3	B2	2	12	2
B3	0	0	0	B3	0	1	0
Kappa value	0.66			Kappa value	0.75		
**Intraobserver agreement (investigator 2)**							
**ME-BLI**				**ME-NBI**			
	**Second Review**				**Second Review**		
First review	B1	B2	B3	First review	B1	B2	B3
B1	62	1	0	B1	65	0	0
B2	2	16	0	B2	5	11	0
B3	0	0	0	B3	0	0	0
Kappa value	0.89			Kappa value	0.78		

SESCC, superficial esophageal squamous cell carcinoma; JES, Japan Esophageal Society; IPCL, intrapapillary capillary loop; ME-BLI, magnifying endoscopy with blue light imaging; ME-NBI, magnifying endoscopy with narrow band imaging.

## Data Availability

The data that support the findings of this study are available from the corresponding author, W.H., upon reasonable request.

## Abbreviations

ER, endoscopic resection; SESCC, superficial esophageal squamous cell carcinoma; T1a-EP/LPM, tumors confined to the epithelium or lamina propria mucosa; T1a-MM/T1b-SM1, tumors invading into muscularis mucosa or submucosa ≤200 μm; T1b-SM2, tumors invading into submucosa >200 µm; IPCL, intrapapillary capillary loop; JES, Japan Esophageal Society; NBI, narrow-band imaging; ME-NBI, magnifying endoscopy with narrow-band imaging; BLI, blue light imaging; ME-BLI, magnifying endoscopy with blue light imaging; PPVs, positive predictive values; NPVs, negative predictive values; CRT, chemoradiotherapy; SD, standard deviation; SE, sensitivity; SP, specificity.
